# Action of the Middle Georgia Medical Society

**Published:** 1875-03

**Authors:** Geo. M. McDowell, R. L. Roddy, L. B. Alexander, K. P. Moore, B. F. Chambliss

**Affiliations:** Forsyth, Ga.; Forsyth, Ga.; Forsyth, Ga.; Forsyth, Ga.; Forsyth, Ga.


*Action of the Middle Georgia Medical Society.*

FORSYTH, GA., January 29, 1875.

To the Secretary and Board of Censors of the Medical Association of Georgia:

GENTLEMEN—The undersigned, members of the Middle Georgia Medical Society, were appointed at the last regular meeting of the Society, held at Forsyth, Ga., January 20, 1875, as a committee to inform your body of the action of this Society in the case of Dr. John G. Westmoreland, of Atlanta, Ga., who was an honorary member of this Society, and is also a member of your body, who was charged with a violation of the Code of Ethics of the American Medical Association, and with conduct detrimental to the honor and usefulness of the profession; and who having refused to make apology for such offense, and having attempted to defend his conduct as herein set forth, was expelled from this Society by the unanimous vote of its members. The Committee was further instructed to charge the said Dr. J. G. Westmoreland before your body with a violation of the Code of Ethics, and with conduct greatly detrimental to the honor and usefulness of our profession, and with contemptuous disregard of the judgment and dignity of this Society, as shown in the specifications hereinafter given.

Very respectfully,
GEO. M. MCDOWELL, M.D.,
R. L. RODDY, M.D.,
L. B. ALEXANDER, M.D.,
K. P. MOORE, M.D.,
B. F. CHAMBLISS, M.D.

We publish the above at the request of friends. The "preamble and resolutions" upon which this Society acted in the expulsion of Dr. J. G. Westmoreland, the specifications and testimony, have been presented to the Board of Censors for its action, with the request that Dr. J. G. Westmoreland, of Atlanta, Ga., be held guilty of the violation of the Code of Ethics, and that he be dealt with accordingly. This Society is composed of seventy or eighty of the best physicians of several of the surrounding counties, and is working on the highest code of Medical Ethics, and will not tolerate irregularity or insubordination to legitimate authority.

